# Strain Rate Sensitivity and Constitutive Law of Closed-Cell PVC Foams under Shock

**DOI:** 10.3390/ma16144995

**Published:** 2023-07-14

**Authors:** Bin Xue, Yong Zhou

**Affiliations:** College of Civil Engineering, Tongji University, Shanghai 200092, China; xuebi@tongji.edu.cn

**Keywords:** close-cell PVC foam, entrapped gas, shock, FEA, inertial effect

## Abstract

The mechanisms of the strain rate dependence of closed-cell PVC foams under shock were numerically studied based on a cell-based model combined with the Coupled Eulerian–Lagrangian (CEL) method in this paper. The strain rate effect of the base material and the entrapped gas effect were focused. The results show that the strain rate effect of the base material has a significant influence on the stress magnitude in the regions before and after the shock front, and the entrapped gas mainly affects the velocity field. Both the strain rate effect of the base material and the entrapped gas have a notable influence on the strain distribution. Taking PVC foam with a relative density of 0.07 as an example, the strain rate effect of the base material will increase the impact stress by 45% and reduce the impact strain by 0.04. The entrapped gas will reduce the impact strain by 0.18, and its effect on the impact stress can be ignored. Finally, two constitutive laws considering the strain rate effect and entrapped gas effect were proposed and compared for the PVC foam under shock with one based on the Hugoniot relationship and the other based on the D-RPH model.

## 1. Introduction

Foams have been widely used mainly as core materials in aerospace, aeronautical, marine, automobile and civil engineering for their high specific strength and stiffness, low self-weight and excellent energy absorption ability [1]. In practice, the components consisting of foam cores have a high possibility of suffering impacts, which can be further categorized as transitional dynamic and shock regimes depending on impact speed or nominal strain rate, i.e., impact speed divided by sample gauge length [2]. With the increase of the impact speed, the forces at the impact end and the stationary end become more and more different due to the macro-inertial effect in the axial direction. In the shock regime, a strong discontinuity of macroscopic properties, e.g., stress and strain, is observed across the shock front, which is the planar cross-sectional surface separating the crushed and the uncrushed cells and propagating along the axis of the specimen with time. Moreover, sequential layer-wise collapse rather than nucleation and propagation of shear bands is the typical shock failure mode of foams under shock.

Under impact, foams show more or less stress enhancement, namely the strain rate effect. Extensive investigations have been carried out to understand the mechanisms of strain rate sensitivity, such as on aluminum foams and on polymeric foams, e.g., polyurethane foam [3,4], polystyrene foam [3,5], polypropylene foam [6] and PVC foam [7,8,9,10,11,12]. Due to the strong strain rate sensitivity of solid PVC, the PVC foams are also strain rate dependent.

It should be noted that the local deformation of foams under high-speed impact is hard to be measured no matter whether the digital image correlation (DIC) or the high-speed camera technique is used [13,14,15,16] and the measurable deformation is limited to very small magnitude, e.g., global strain ~0.15 [16]. It is also hard to distinguish the macro-inertia effect from the strain rate effect of the base material in an experiment. So, many numerical models have been established to investigate the dynamic properties of foams under shock, e.g., 2D [17] and 3D [18,19] Voronoi cell-based models, X-ray micro-computed tomography (XCT) image-based models [20,21,22,23]. Although the XCT imaged-based model is alleged as the closest to real foams, some errors are introduced during the image processing and meshing. For example, the relative density of the numerical model is 11–21% larger than that of the scanned sample [20,22]. Therefore, Voronoi cell-based model, which balances the accuracy and computational expense, is preferred in the numerical analysis of foams.

From the above-mentioned research, it is generally accepted [2] that the strain rate dependence of aluminum foam is the consequence of the micro-inertia effect of cell walls, gas pressure in the cells, strain-rate sensitivity of the base material and shock wave effect for high impact speed although the former two effects are still in controversy.

Some researchers [24,25] thought that the micro-inertia effect could be an important factor to cause the macroscopic rate sensitivity of foams, for the delay of the buckling by micro-inertia prolongs the uniaxial compression of cell walls. Tan et al. [26] credited the strain-rate dependence of the plastic collapse stress to the micro-inertial effect below the critical velocity and to the macro-inertial effect above the critical velocity, i.e., in the shock regime, respectively. The Type II structure [27], i.e., a pair of slightly bent elastic–plastic struts, was generally used to explain the micro-inertia effect. Gao et al. [28] found that a smaller initial rotation angle of the struts led to a larger equivalent mass.

However, through the finite element analysis (FEA) on foams with randomly oriented cell walls, it is found [2,29] that the transverse to longitudinal acceleration ratio of a cell wall decreases with the increase of loading rate, combined with the observation that the local strain rates measured at mesoscopic scale are at least one order of magnitude greater than the global strain rates applied on the specimen [15], the contribution of micro-inertia effect is believed negligible in the shock regime. This is to some extent in consistence with the conclusion of Zou et al. [30], who found that a single Type II structure was more sensitive to transverse inertia than a chain of Type II structure and the latter was more similar to the multi-layered structure of foams.

As to the influence of gas within foam cells, the interaction of cell walls and the gas was assumed to enhance the strength of foams under impact [31]. From the FEA of a regular Kelvin foam model combined with the fluid cavity model to consider the cell air, Mills et al. [32] attributed the strength enhancement of impacted low-density closed-cell polyethylene foam to a combination of the face-to-face contact and gas pressure for high engineering strain range (>0.6–0.8) and to the gas pressure only for low engineering strain range (<0.6). However, the gas effect is regarded as negligible in many studies on aluminum foams [16,23,29,33,34,35,36,37]. The gas pressure was believed to become significant only when the cells had been crushed, but before that, the applied compression had been transferred to adjacent cells whose cell pressures were still low, so the gas effect could not enhance the foam strength until all the cells had been crushed and the densification started.

Sandwich structures with PVC foam core are superior to those with aluminum foam core in the energy absorption capacity because the PVC foam has a much larger absorption energy/density ratio than the aluminum foam [38]. Unfortunately, very few studies touch upon the dynamic response of the PVC foam under shock. For example, although the strain rate as high as 4000 s^−1^ was used by Tagarielli et al. [8] to measure the uniaxial dynamic compressive responses of PVC foams, the specimen thicknesses in the axial direction were only 4.89 mm and 4.86 mm for Divinycell H100 and H250, respectively, so that the corresponding impact speed was less than 20 m/s, under which the shock wave propagation only played a minor role. The mechanical responses of the PVC foam under shock were numerically investigated in this paper and the mechanisms of strain rate effect, i.e., the micro-inertia effect of cell walls, gas pressure in the cells, strain-rate sensitivity of the base material and shock wave effect, were clarified. Since the cell structure, which varied from each other among different types of foams manufactured by different processes, had a significant effect on the strain-rate sensitivity, this study focused on the H-series PVC foam from the Diab Group, whose cell structure had been well calibrated in previous research [39,40]. In this study, the Voronoi cell-based model was used to characterize the geometric characteristics of the closed-cell PVC foam and the CEL model in ABAQUS was employed to consider the solid–gas interaction of the PVC foam under different impact velocities.

The remainder of this paper is organized as follows. Section 2 details the cell-based FEA model of the PVC foam. The mechanical responses of the PVC foam under quasi-static compression and impact of different speeds are compared in Section 3. Section 4 emphasizes the influences of the strain rate effect of the base material and the entrapped gas pressure to the strain rate effect of the foam under shock. Section 5 presents a constitutive model for the PVC foam under shock. Finally, conclusions are given in Section 6.

## 2. FEA Model

The FEA model of the closed-cell PVC foam was produced following the procedures in [39]. A group of spheres, whose volumes obey the Gamma distribution measured by XCT, were packed compactly using the advancing front method in a container of larger dimensions than those of the model. Then, the container was divided into polyhedrons based on the positioned spheres with the Laguerre tessellation. Finally, the thickness was assigned to the polyhedron faces and the model was cut from the container. For the foams with the relative density $\rho^{*}/\rho_{s}$ = 0.07, 0.14 and 0.21, where $\rho^{*}$ and $\rho_{s}$ were the density of the foam and the density of the base material. The average equivalent diameter of the cells without considering the thickness was 360 µm and the thicknesses with relative density from low to high were 11.5 µm, 23.0 µm, and 34.5 µm, respectively.

The interaction between the entrapped gas and cell walls was simulated with the Coupled Eulerian–Lagrangian (CEL) method [41], in which the entrapped gas and cell walls were handled by the Eulerian and Lagrangian elements, respectively. Due to the short impact time, the heat transfer was constrained, thus an adiabatic state was assumed for the gas during the impact process [42]. The equation of state of an ideal gas was assumed for the entrapped gas with the corresponding parameters shown in Table 1. Bouix et al. [6] experimentally confirmed that the gas can be effectively retained within the cells of polypropylene foam under high-speed impact. So, the segmentation of the cell walls and the consequent gas flow were ignored. A perfect elastoplastic constitutive law was adopted for the base material with an elastic modulus of 2700 MPa [43]. Because the impact is transient, the influence of temperature increase on the mechanical properties of the base material was neglected. The yield strength of the base material was assumed proportional to the logarithm of the strain rate (Equation (1)).

(1) $$\sigma_{y}=92.1+10.05log\dot{\varepsilon }$$

**Figure 1:**
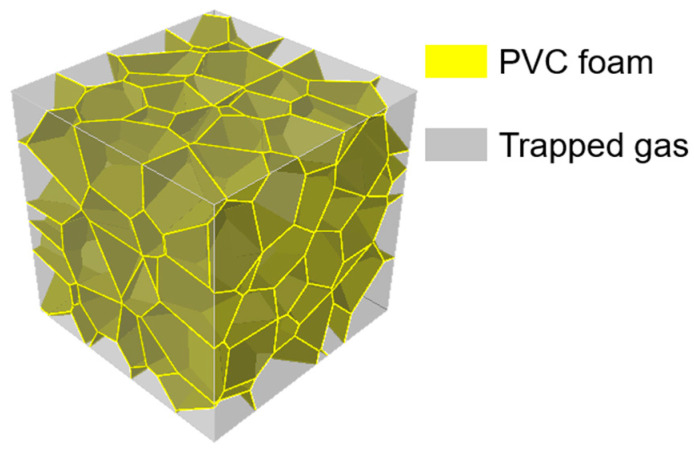
FEA model.

The density and the Poisson’s ratio of the base material were set to be 1400 kg/m^3^ and 0.38, respectively. The established FEA model is shown in Figure 1.

The numerical simulations were performed using the Abaqus/Explicit [41]. The cell walls were discretized using shell elements SR3 and SR4 and the Eulerian domain was meshed using EC3D8R elements. The average mesh size was determined to be 0.02 mm by mesh sensitivity analysis. A frictionless general contact was assumed between the gas domain and the shell elements.

The three-dimensional foam specimen was sandwiched between two rigid plates. During the loading process, the upper plate moved downwards at a constant velocity as the loading (impact) end, while the lower plate was fixed as the supporting end. Surface-to-surface contact was assumed for the contacts between the plates and foam and between cell walls with a tangential friction coefficient of 0.2 and a hard contact in the normal direction, in which the separation after contact was allowed.

As to the dimensions of the FEA model, a 2 mm cube was selected for the following reasons: (1) the 2 mm cubic numerical specimen was able to simulate the quasi-static compression accurately [40]; (2) the mechanical behavior of foams became more locally under impact and thus reduce the requirement on the representative element volume; (3) the average equivalent diameter of the studied PVC foams was 360 μm, meeting the suggestion of Andrews et al. [44], i.e., at least five cells along the shortest dimension, to eliminate possible size effect. Using an average mesh size of 0.02 mm for division, the 2 mm RVE has 132,704 meshes, including 121,868 S4R meshes and 10,836 S3R meshes.

The compressive stress in the subsequent sections was defined as the reaction force of the rigid plate divided by the original surface area of the model. The compressive strain was defined as the displacement of the upper plate divided by the total length of the FEA model along the loading direction.

## 3. Comparison of Quasi-Static Compression and Impact with Medium- and High-Speed

**Figure 2:**
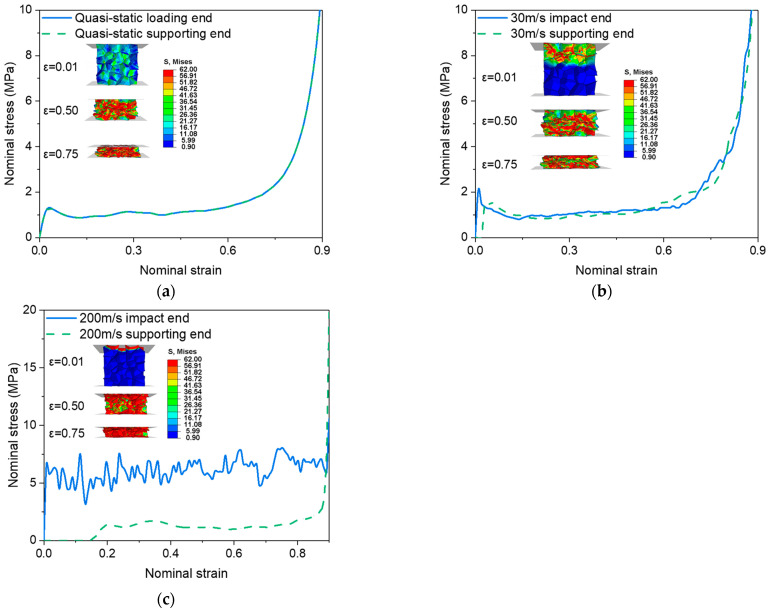
Deformation modes and stress–strain curves at both the impact and support ends under uniaxial loading neglecting the strain rate effect of the base material and the entrapped gas effect. (**a**) Quasi-static; (**b**) $V_{i}=$ 30 m/s; (**c**) $V_{i}=$ 200 m/s.

In this section, the FEA were conducted on the PVC foam with the relative density $\rho^{*}/\rho_{s}$ = 0.07 under quasi-static compression (strain rate of 0.001 s^−1^), medium-speed impact at $V_{i}=$ 30 m/s and high-speed impact at $V_{i}=$ 200 m/s. The strain rate effect of the base material and the entrapped gas effect were neglected, whereas the macro- and micro-inertia were automatically considered by the explicit algorithm of the Abaqus/Explicit [41]. To improve the computational efficiency in the quasi-static loading, the model was scaled in mass, but the kinetic energy/internal energy ratio was always kept less than 5%. It is obvious from Figure 2 that the deformation modes of the PVC foam under uniaxial loading of various speeds are different. The deformation mode under quasi-static compression is characterized by random shear bands, whereas the layer-by-layer crushing happens under the 200 m/s high-speed impact. The deformation mode under the 30 m/s medium-speed impact is a mixture of those under the quasi-static compression and high-speed impact.

In addition, the delayed equilibrium between the loading end and the supporting end becomes more and more apparent with the increase in the loading speed. Under the quasi-static compression, the stress–strain curves at the two ends almost coincide, indicating negligible inertial effect. At 30 m/s, the compressive stresses at the supporting end lag behind those at the loading end due to the inertial effect. But because the inertia effect is not significant, the stresses at the two ends tend to synchronize once the supporting end has been stressed. Consequently, the stress–strain curve at the loading end can be used to describe the overall compression behavior of the foam. However, at 200 m/s, the inertial effect is dominant, leading to much higher stresses and more severe oscillations at the loading end compared to the supporting end. Therefore, local stress–strain states rather than the stress–strain curve at either end should be used to characterize the dynamic responses of the foam [19].

**Figure 3:**
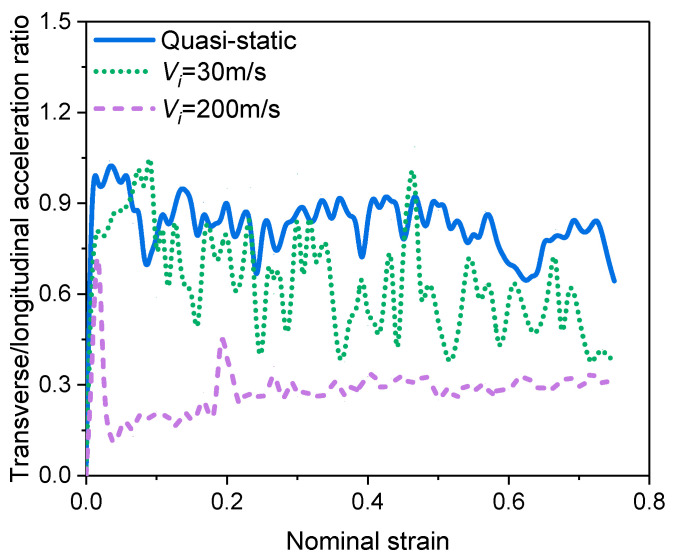
Transverse/longitudinal acceleration ratio.

In order to investigate the effect of micro-inertia effects on the mechanical properties of impacts, the method of ref. [29] was referred to by extracting the transverse and longitudinal accelerations of all nodes and taking the ratio of the transverse and longitudinal accelerations as a quantitative evaluation index. The results of the transverse acceleration/longitudinal acceleration at different velocities are shown in Figure 3. It can be found that the ratio is much smaller than that of a typical type II structure and decreases with increasing impact velocity, indicating that the micro-inertia effect of the foam material is weak.

## 4. Mechanical Response to High-Speed Impact Considering Entrapped Gas Effect and Strain Rate Effect

Since it has been concluded from Section 3 that in the shock regime, the macro-inertia effect is dominant and the micro-inertia effect is marginal, the influences of the other two strain rate dependence mechanisms, i.e., the strain rate effect of the base material and the entrapped gas effect, to the PVC foams of different densities under different impact speed will be investigated in this section.

To facilitate the comparison of the two mechanisms, four load cases (LCs) were designed. None of the mechanisms were considered in LC1, both of the mechanisms were included in LC4, LC3, and LC2 considered only one mechanism, i.e., the strain rate effect of the base material and the entrapped gas effect, respectively.

### 4.1. Stress at the Impact and Supporting Ends

**Figure 4:**
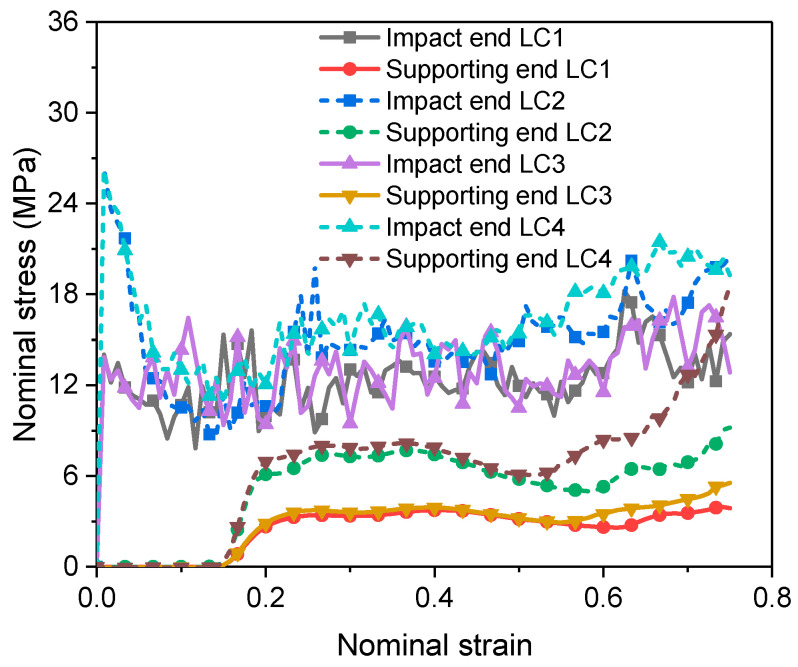
Stress–strain curves at the two ends of the PVC foam ($\rho^{*}/\rho_{s}=$ 0.14) under an impact of 200 m/s.

The stresses at the impact end and the supporting end are important parameters to determine the impact constitutive behavior [45]. Taking $\rho^{*}/\rho_{s}=$ 0.14 and $V_{i}=$ 200 m/s as an example, the stresses at the two ends in the four LCs are compared in Figure 4.

**Figure 5:**
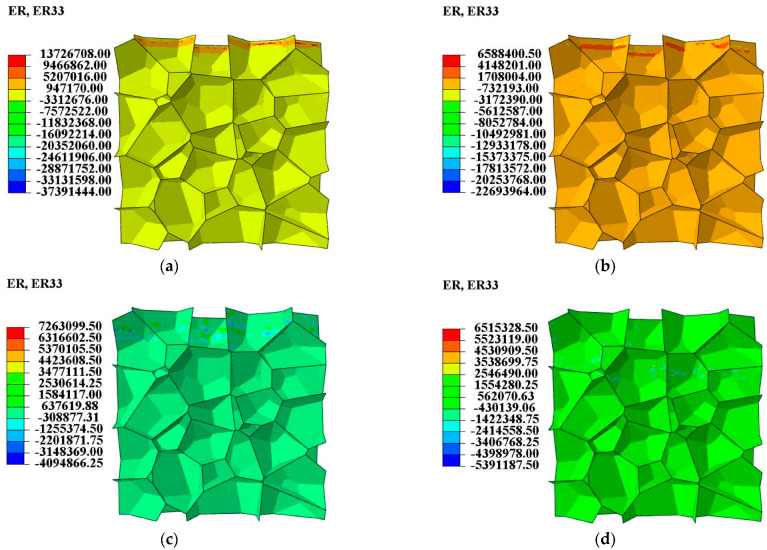
Strain rate distribution of LC2 at different nominal strains in the original reference configuration. (**a**) Nominal strain = 0.01; (**b**) Nominal strain = 0.02; (**c**) Nominal strain = 0.10; (**d**) Nominal strain = 0.30.

It can be seen from Figure 4 that the yield strength (first peak stress) at the impact end in LC2 and LC4 is much higher than that in LC1 and LC3 due to the strain rate effect of the base material. However, as the shock wave propagates downwards at a constant speed, the strain rate increment cannot be sustained although the global strain still increases, and a gradual decrease of the strain rate behind the shock front is observed (Figure 5). This explains the sharp drop of the stress after its first peak in LC2 and LC4. To make the strain rate distribution clearer seen, the original reference configuration is adopted in Figure 5.

The time when the stress reaches the supporting end is nearly a constant in all LCs, indicating that the strain rate effect of the base material and the entrapped gas effect have negligible influence on the shock wave propagation.

**Figure 6:**
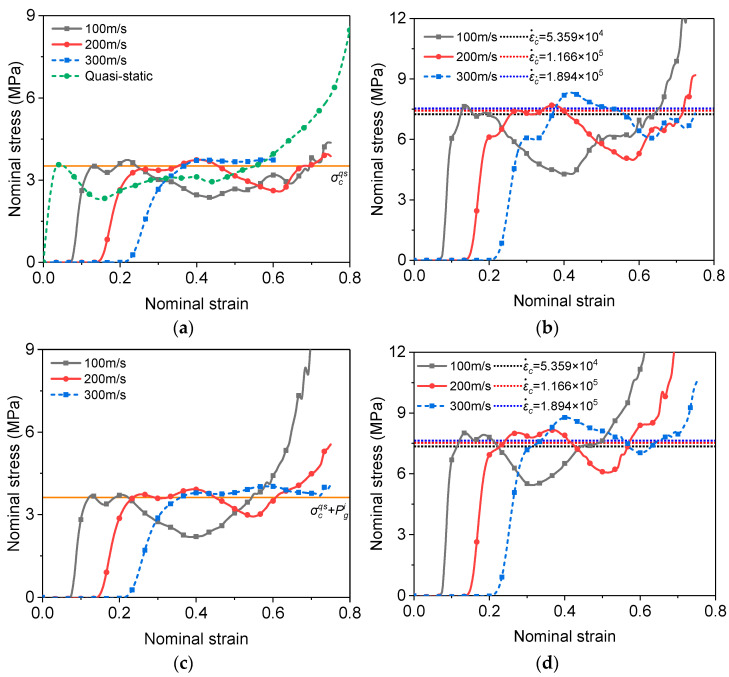
Stress–strain curves at the supporting end for the foam with $\rho^{*}/\rho_{s}$ = 0.07 impacted at three different velocities. (**a**) LC1; (**b**) LC2; (**c**) LC3; (**d**) LC4.

As to the stress at the supporting end, the influences of the density ($\rho^{*}/\rho_{s}$ = 0.07, 0.14 and 0.21) and the impact speed ($V_{i}=$ 100, 200 and 300 m/s) were also studied in addition to the mechanisms of strain rate dependence. Figure 6 shows the stress–strain curves at the supporting end for the foam with $\rho^{*}/\rho_{s}$ = 0.07 impacted at three different velocities. It can be seen that the yield strengths in LC1 (Figure 6a) at the three impact speeds are not much different from that under quasi-static loading. Therefore, when the strain rate effect of the base material and the entrapped gas effect are neglected, the quasi-static compressive yield strength $\sigma_{c}^{qs}$ (the horizontal line in Figure 6a) can be used to characterize the foam in front of the shock front, as suggested by Xu et al. [17,45]. When only the entrapped gas effect is included (Figure 6c), its influence on yield strength is small. However, after the initial gas pressure $P_{g}^{i}$ (the ambient pressure in Table 1) has been introduced, the sum of $\sigma_{c}^{qs}$ and $P_{g}^{i}$ (the horizontal line in Figure 6c) is more consistent with the stress at the supporting end predicted by the FEA. The reason to choose the ambient pressure is that the gas entrapped in the cells in front of the shock front is not significantly compressed.

**Figure 7:**
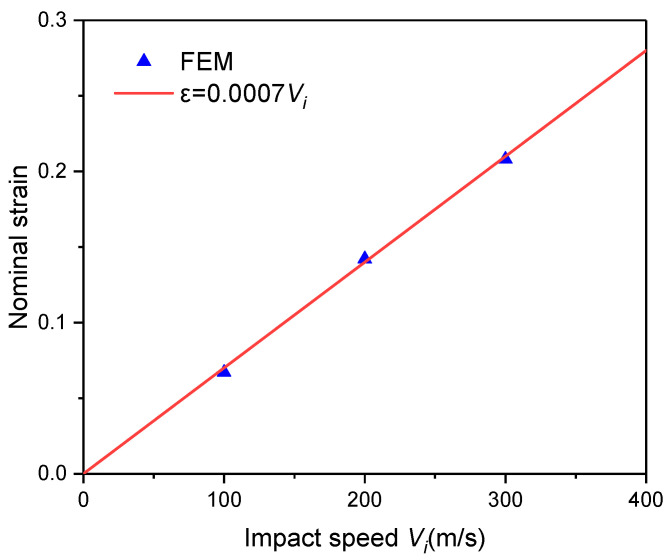
Relationship between the nominal strain and impact speed in LCxxx when the stress reaches the supporting end.

When the strain rate of the base material is considered, it is evident that the stress is greatly increased (Figure 6b,d). The authors suggest that the sample gauge length is replaced by the specimen thickness at the time when the stress reaches the supporting end to give a more realistic strain rate and is called calculated strain rate ${\dot{\varepsilon }}_{c}$, which will be used in the forthcoming prediction equation for the compressive yield strength of the foam. For $V_{i}=$ 100, 200 and 300 m/s in the present study, the specimen thickness and the corresponding calculated strain rates when the stress reaches the supporting end are 1.866 mm, 1.716 mm, 1.584 mm and 5.359 × 10^4^, 1.166 × 10^5^, 1.894 × 10^5^, respectively. By extracting the nominal strain corresponding to the stress reaching the support end at different impact speeds, it can be found that the nominal strain is basically linear with the impact speed (Figure 7).

**Figure 8:**
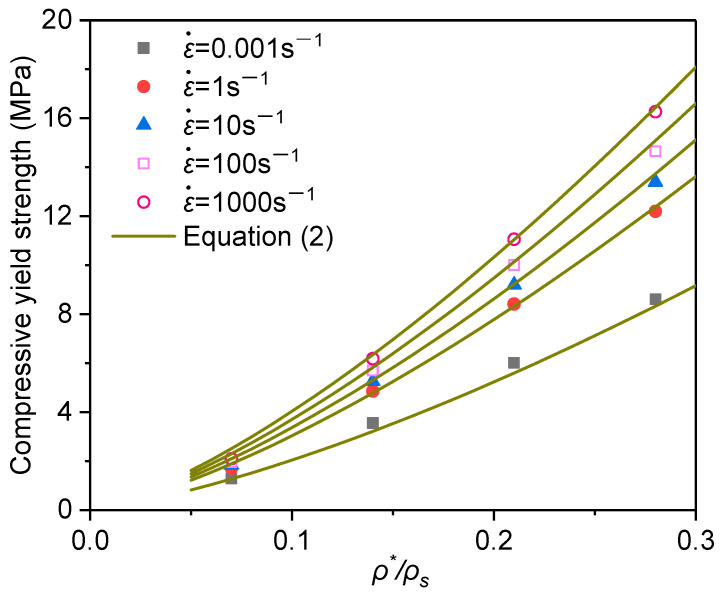
Compressive yield strength of the PVC foam with the relative densities under the impacts of different speeds.

The uniform stress state in the region ahead of the shock front indicates that the inertial effect can be neglected. Simulations of the same PVC foam under uniaxial compression of low to medium strain rate, in which the inertia effect is secondary and the strain rate effect of the base material is considered, show that the compressive yield strength of the PVC foam is proportional to the logarithm of strain rate as described by Equation (2) [46].

(2) $$\sigma_{c}\left(\dot{\varepsilon }\right)=\sigma_{ys}[1+\lambda_{2}log10\left(\frac{\dot{\varepsilon }}{{\dot{\varepsilon }}_{0}}\right)]\left[C_{3}{\left(\phi \frac{\rho^{*}}{\rho_{s}}\right)}^{3/2}+C_{4}(1-\phi )\frac{\rho^{*}}{\rho_{s}}\right]$$

where $\sigma_{ys}$ is the quasi-static yield strength of the base material (62 MPa), $C_{3}=0.8$7, $C_{4}=0.82$, ϕ=0.87 and $\lambda_{2}$ = 0.162 are the fitting parameters, $\rho^{*}/\rho_{s}$ is the relative density an ${\dot{\varepsilon }}_{0}$ is the strain rate corresponding to the quasi-static condition (0.001 s^−1^). The fitting results are shown in Figure 8.

Herein, Equation (2) is extended to higher strain rate. The yield strength predicted with the suggested nominal strain rate by Equation (2) (the horizontal lines in Figure 6b,d) agrees reasonably well with the FEA result for all impact speeds.

**Figure 9:**
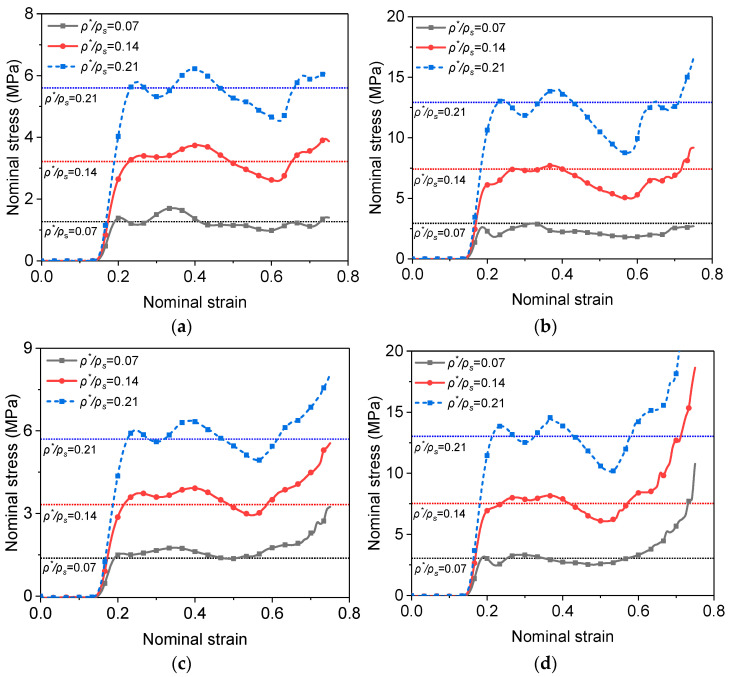
Stress–strain curves at the supporting end for the PVC foams with different relative densities impacted at 200 m/s. (**a**) LC1; (**b**) LC2; (**c**) LC3; (**d**) LC4.

Figure 9 shows the stress–strain curves at the supporting end for the PVC foams of different relative densities at the speed of 200 m/s. It can be found that the relative density has a significant impact on the compressive stress at the support end. But the yield strength also follows the relationship with relative density and nominal strain rate expressed by Equation (2) under high-speed impact. The influence of the entrapped gas on the stress at the supporting end is marginal.

### 4.2. Velocity Field Distribution

To investigate the mechanical properties of the PVC foam under shock, it is crucial to obtain the velocity field along the impact direction. An averaging method [47] should be used to mitigate the errors caused by the irregular micro-structure of the foam. In this study, the specimen was evenly divided into 10 segments along its length, resulting in 11 cross-sections $\left(X_{i},\;i=1,\;2,\;\ldots ,11\right)$ to measure the velocity with the impact end being $X_{1}$ and the supporting end $X_{11}$. The velocity at $X_{1}$ remained constant and was equal to the loading rate, while the velocity at $X_{1}$ remained zero. For other $X_{i}$, the velocity at each cross-section was calculated as the mean of all nodal velocities within the range of X−d/2 to X+d/2, where d was the average cell diameter. This is because the thickness of the shock front in foams is generally assumed to be one cell size and barely dependent on the crushing velocity and relative density [2,26,48,49].

**Figure 10:**
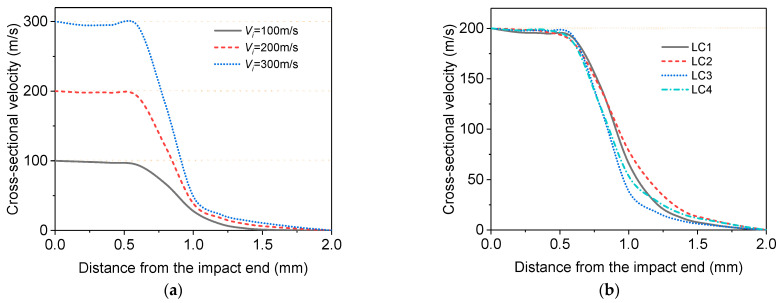
Velocity fields at the nominal strain of 0.4 for: (**a**) different impact speeds, $\rho^{*}/\rho_{s}$ = 0.14, LC1; (**b**) different LCs, $\rho^{*}/\rho_{s}$ = 0.14, $V_{i}=$ 200 m/s.

The velocity distribution under shock is shown in Figure 10, where Figure 10a describes the velocity fields of the PVC foam with $\rho^{*}/\rho_{s}$ = 0.14 impacted at the speeds of $V_{i}=$ 100, 200 and 300 m/s for LC1, and Figure 10b presents the velocity fields of the foam with $\rho^{*}/\rho_{s}$ = 0.14 at the impact speed of 200 m/s for all four LCs. Note: LC stands for Load case, normalized distance is the distance of the Lagrangian position from the impact end.

From Figure 10a, it can be observed that for the same LC, e.g., LC1, the velocity in the region behind the shock wave is close to the impact speed, whereas the velocity in the region in front of the shock wave is almost zero. At the shock front, the velocity field distribution exhibits a jump, which becomes significant with the increase of the impact speed. Figure 10b shows the influences of different strain rate mechanisms on the velocity distribution along the specimen length. The comparison of LC1 and LC3, both of which ignore the strain rate effect of the base material, shows that the presence of entrapped gas causes the shock front to move towards the supporting end. The reason is that when the entrapped gas is compressed, it rapidly transfers the pressure to adjacent cells and the deformation of the next layer of cells leads to the advance of the shock front. For LC1 and LC2, where the entrapped gas effect is neglected, it can be found that the strain rate effect of the base material mainly affects the velocity field ahead of the shock front, i.e., reducing the decay rate of the velocity, but has little influence on the position of the shock front, where the velocity drops to half of the impact speed [45].

**Figure 11:**
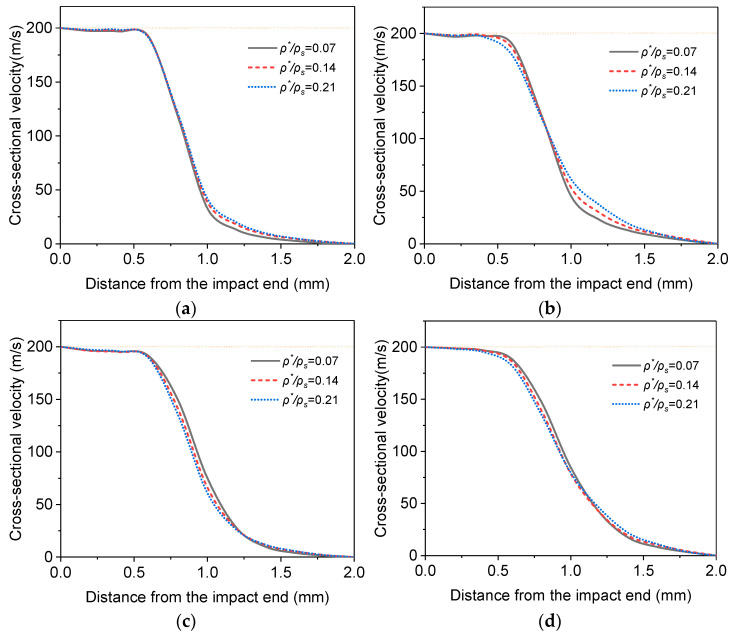
Velocity fields at the nominal strain of 0.4 for the foams with different densities impacted at 200 m/s. (**a**) LC1; (**b**) LC2; (**c**) LC3; (**d**) LC4.

Because the thickness of a cell wall determines the stiffness and thus the deformation of the cell wall, the velocity fields of the foams with different relative densities, which are also dependent on cell wall thickness, at the impact speed of 200 m/s are analyzed for different LCs and shown in Figure 11 at the nominal strain of 0.4.

It is clear from Figure 11 that the relative density has nearly no influence on the velocity distribution when neither the entrapped gas effect nor the strain rate effect of the base material is considered (LC1). If only the strain rate effect (LC2) of the base material is considered, the position of the impact front is almost unaffected. At the same time, the greater the relative density of the position after the shock front, the faster the speed decreases. The greater the relative density of the position before the shock front, the slower the speed decreases. When only the entrapped gas effect is considered (LC3), because the deformation of the cells at the shock front, which is approximately in the thickness of one average cell size, becomes more significant for lower relative density, the shock front for higher relative density is closer to the impact end.

### 4.3. Location of the Shock Front and Velocity of Shock Wave

**Figure 12:**
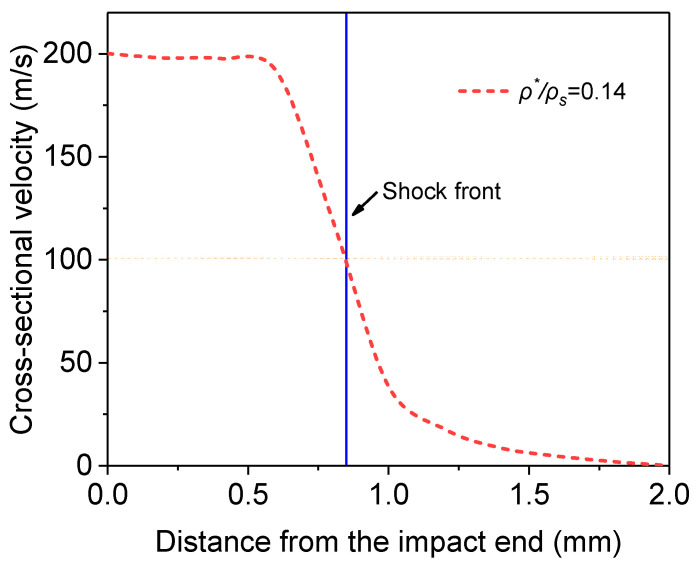
Determination of the location of the shock front.

After the cross-sectional velocity distribution has been obtained, the position of the shock front can be determined. Currently, there are mainly two methods to decide the shock front position. One is to use the Lagrangian position where the velocity gradient reaches its maximum [37], and the other is to directly use the cross-sectional position where the velocity drops to half of the impact speed [45]. The two methods give nearly the same shock front position, so the second method is adopted in this paper (Figure 12).

**Figure 13:**
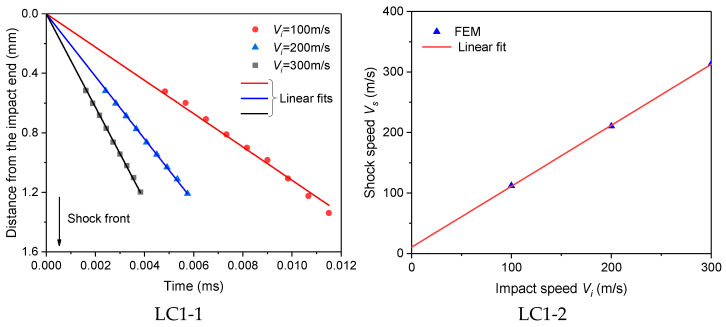

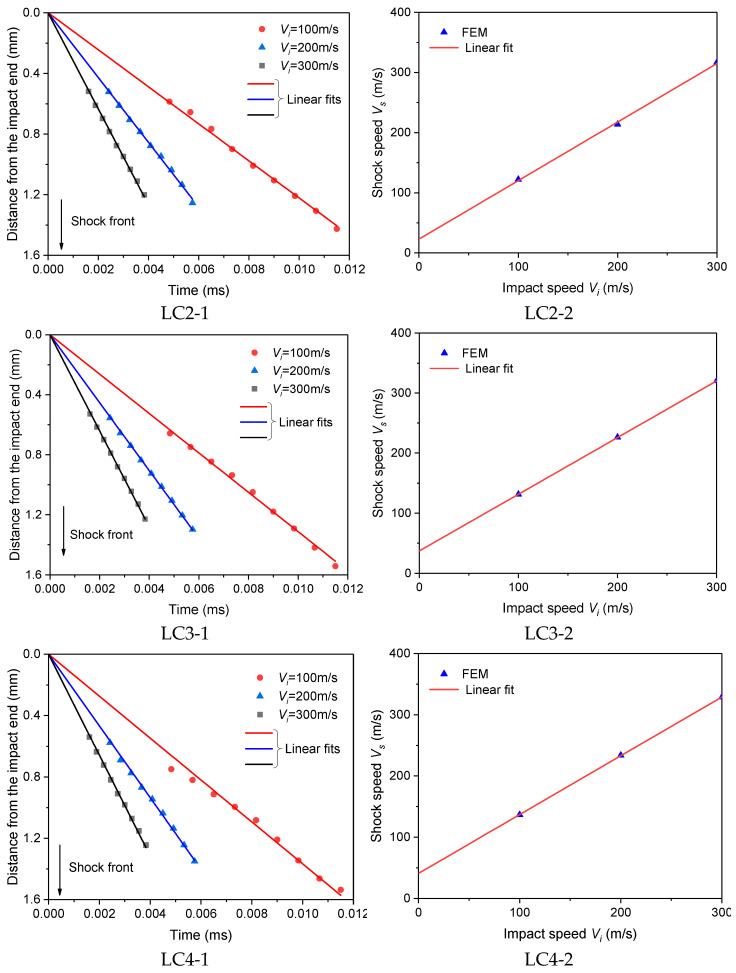
Movement of the shock front (LCx-1) and its velocity vs the impact speed (LCx-2).

The movement of the shock front and its velocity vs. the impact speed for the four LCs under different impact speeds are shown in Figure 13 with $\rho^{*}/\rho_{s}$ = 0.14 as an example. It can be found that for all LCs, the locations of the shock front and the loading time can be approximately fitted to a straight line, whose slope gives the shock wave velocity $V_{s}$. And the shock wave velocity has a linear relationship with the impact speed $V_{i}$ as

(3) $$V_{s}=V_{r}+SV_{i}$$

where $V_{r}$ is the reference velocity, i.e., the intercept of $V_{r}$, and S is the material parameter. Their values for the foams of different velocities in the four LCs are displayed in Table 2.

As can be seen from Table 2, the slope S is close to 1 and varies very little. However, the reference velocity $V_{r}$, which is very close to the critical impact velocity for shock initiation [2], is significantly affected by the strain rate effect of the base material and the entrapped gas, and both mechanisms have a reinforcing effect on it.

### 4.4. Stresses before and after the Shock Front

**Figure 14:**
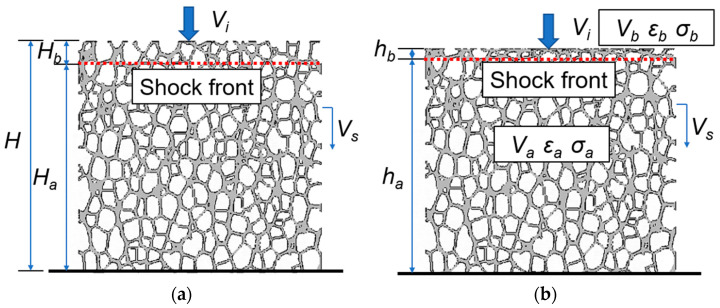
Illustration of the shock wave propagation (adapted from [45]) in (**a**) Lagrangian configuration and (**b**) Eulerian configuration.

As the foam material has the same propagation mode of shock wave as the continuum solid under impact, the conservation law in the continuum solid also applies to the foam material [45]. From the conservation equations for mass, momentum, and energy [50], the following equations can be obtained,

(4) $$V_{b}-V_{a}=V_{s}\left(\varepsilon_{b}-\varepsilon_{a}\right)\;,$$

(5) $$\sigma_{b}-\sigma_{a}=\rho^{*}V_{s}\left(V_{b}-V_{a}\right),$$

where $\rho^{*}$ is the initial density of the foam, $V_{k}$, $\varepsilon_{k},$ and $\sigma_{k}$ (k=a, b) are the velocity, strain and stress in the region in front of (a) and behind (b) the shock front, respectively (Figure 14). The shock strain $\varepsilon_{b}$ is the deformation measured in the crushed zone.

Since the region ahead of the shock front is almost undeformed, the following assumptions can be made, $\varepsilon_{a}\approx 0$, $h_{a}\approx H_{a}$ and $V_{a}\approx$ 0. In the crushed zone behind the shock front, $V_{b}\approx V_{i}$ can be approximated from the velocity field. Therefore,

(6) $$\varepsilon_{b}\approx \frac{V_{i}}{V_{r}+SV_{i}},$$

(7) $$\sigma_{b}\approx \sigma_{a}+\rho^{*}V_{i}\left(V_{r}+SV_{i}\right).$$

Since the mechanical properties ahead of the shock front are assumed uniform, $\sigma_{a}$ is generally taken as the yield strength of the foam at the supporting end, e.g., $\sigma_{a}\approx \sigma_{c}^{qs}$ [45] or $\sigma_{a}\approx {\sigma_{c}^{qs}+P}_{g}^{i}$ [51]. The former expression does not consider the entrapped gas effect, or the strain rate effect of the base material and the latter only considers the entrapped gas effect. However, it can be seen from Figure 6c that the strain rate effect of the base material has an effect on the yield strength of the foam at the supporting end. Therefore, $\sigma_{a}\approx \dot{\sigma_{c}}$ (Equation (2)) is recommended in this study when only the strain rate effect of the base material is included, whereas $\sigma_{a}\approx \dot{\sigma_{c}}+P_{g}^{i}$ when both the strain rate effect of the base material and the entrapped gas effect are considered. It should be noted that $\dot{\sigma_{c}}$ is the compressive yield strength based on Equation (2) and calculated strain rate ${\dot{\varepsilon }}_{c}$ (detailed in Section 4.1).

On the other hand, $\varepsilon_{b}$ and $\sigma_{b}$ can be obtained directly from the FEA, i.e., $\varepsilon_{b}=\left(H_{b}-h_{b}\right)/H_{b}\approx \left(H-h_{a}-h_{b}\right)/\left(H-h_{a}\right)$ and $\sigma_{b}$ being the average reaction force at the impact end divided by the cross-sectional area of the original sample.

**Figure 15:**
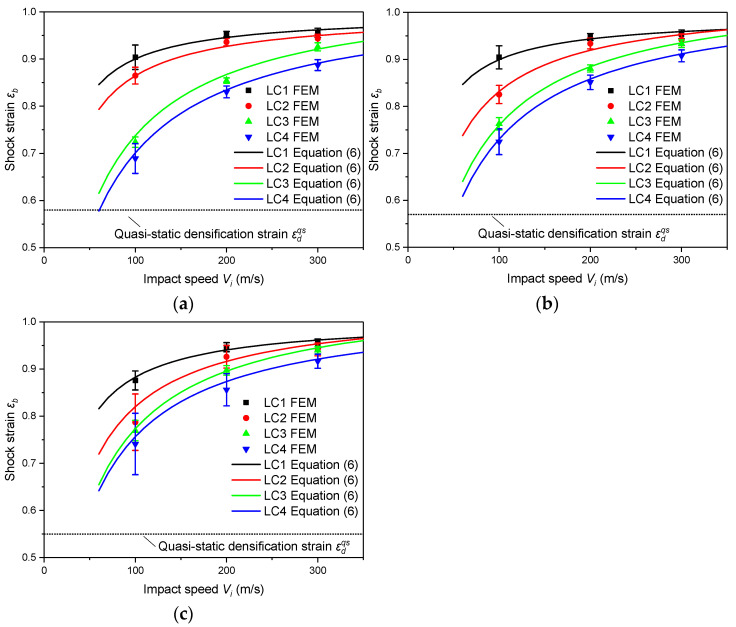
Shock strain in the region behind the shock front. (**a**) $\rho^{*}/\rho_{s}=0.07$; (**b**) $\rho^{*}/\rho_{s}=0.14$; (**c**) $\rho^{*}/\rho_{s}=0.21$.

**Figure 16:**
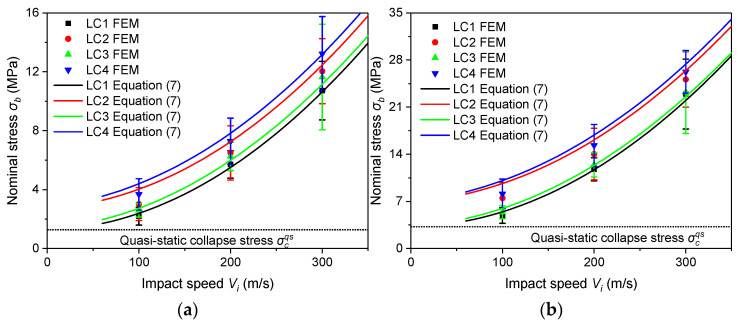

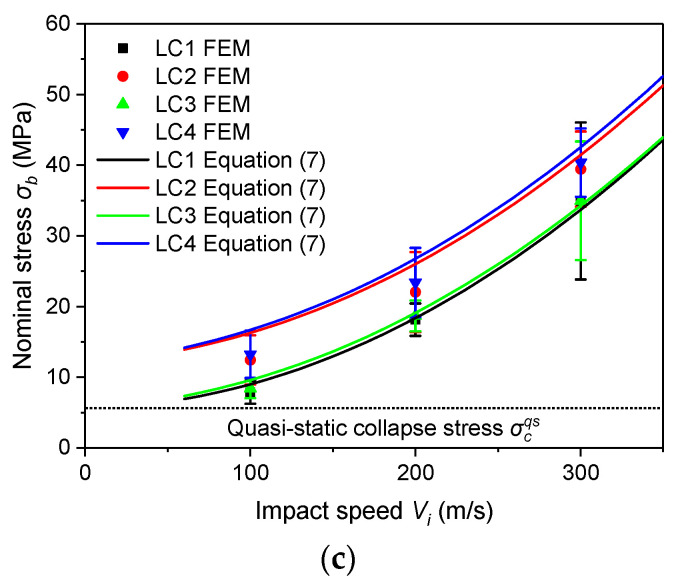
Stresses behind the shock front. (**a**) $\rho^{*}/\rho_{s}=0.07$; (**b**) $\rho^{*}/\rho_{s}=0.14$; (**c**) $\rho^{*}/\rho_{s}=0.21$.

The FEA predicted shock strain and nominal stress are compared with Equations (6) and (7) in Figure 15 and Figure 16, respectively. In order to eliminate the data fluctuation caused by the explicit algorithm, the shock strain $\varepsilon_{b}$ is averaged in a series of measurements, and the error line is used to represent the standard deviation of the measurements under different nominal strains.

It can be found from Figure 15 that $\varepsilon_{b}$ under shock is greater than the densification strain under quasi-static loading. Both the entrapped gas effect and the strain rate effect of the base material reduce $\varepsilon_{b}$ and their influences decrease with the increase of the impact speed. Meanwhile, the entrapped gas has a greater influence than the strain rate of the base material.

Figure 16 shows that Equation (7) can effectively characterize the stress state in the region behind the shock front for the LCs. It is the strain rate effect of the base material that mainly affects $\sigma_{b}$, whereas the entrapped gas has only a slight enhancement. Moreover, the error bar of $\sigma_{b}$ becomes larger with the increase of the impact speed. This is because the higher the impact speed, the larger the stress fluctuation is at the impact end. However, the error does not vary significantly among the four LCs.

## 5. Constitutive Law under Uniaxial Shock

Many constitutive laws have been proposed for foam materials under shock [19,52,53,54]. Herein, two models [19,45] were modified to include the strain rate effect of the base material and the entrapped gas effect and are compared with the FEA results for the four LCs.

In the first model (Equation (8)) [45], $\sigma_{a}$ discussed in Section 4.4 should be used.

(8) $$\sigma_{b}\approx \sigma_{a}+\rho^{*}\varepsilon_{b}{\left(\frac{V_{r}}{1-S\varepsilon_{b}}\right)}^{2}$$

In the second D-R-PH (dynamic-rigid-linear hardening plastic-locking) model (Equation (9)) [19], the quasi-static and dynamic cases can be distinguished by adjusting the fitting parameters *D* and $\sigma_{0}^{d}$ can be assumed to be $\sigma_{a}$ [51].

(9) $$\sigma (\varepsilon )=\sigma_{0}^{d}+\frac{D\varepsilon }{(1-\varepsilon {)}^{2}}$$

**Figure 17:**
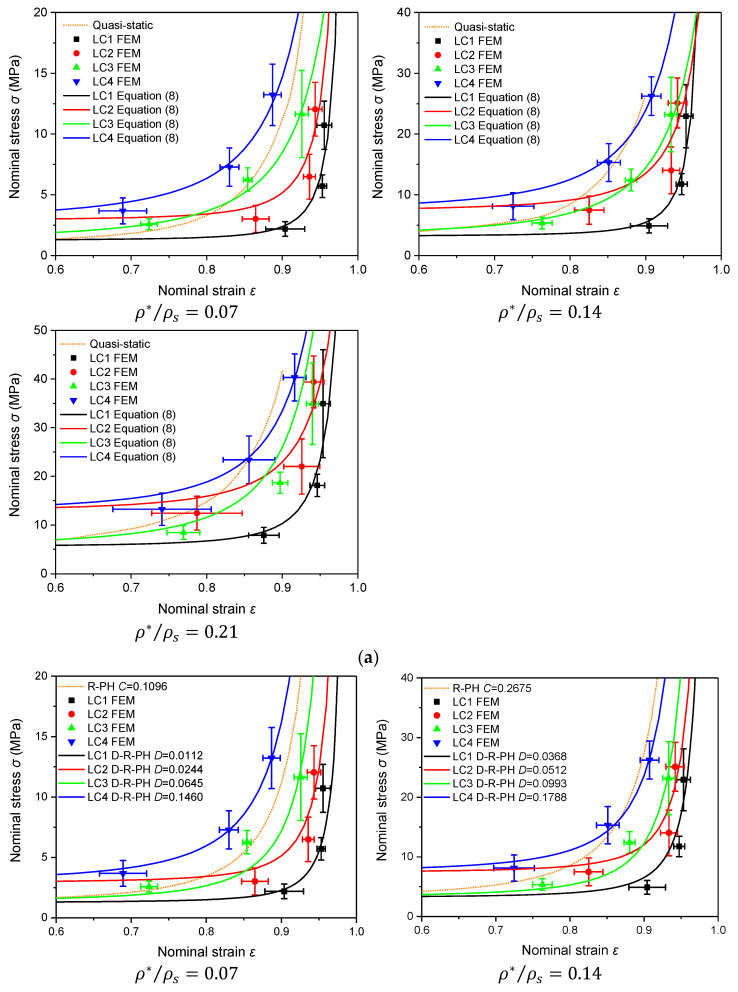

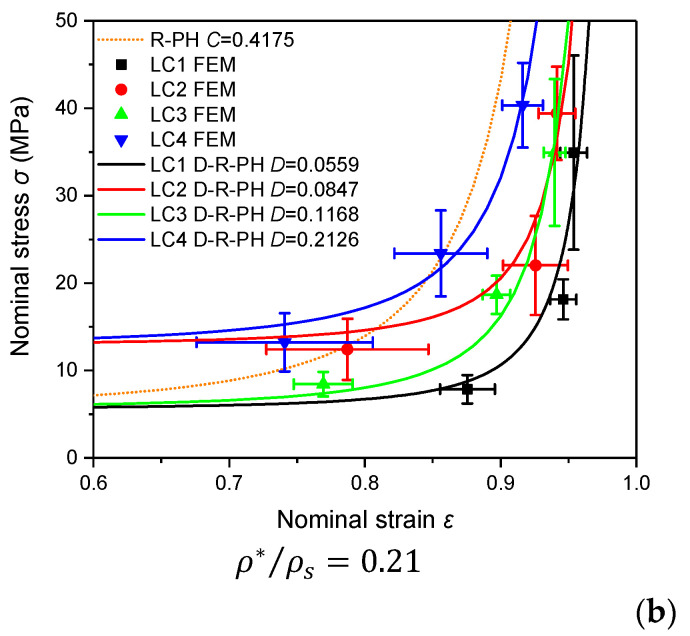
Constitutive laws under impact. (**a**) FEA vs. Equation (8); (**b**) FEA vs. Equation (9).

Taking $\rho^{*}/\rho_{s}$ = 0.14 as an example, Figure 17a,b shows the comparisons of the FEA results with Equations (8) and (9), respectively.

When the stress is plotted against strain, from low to high corresponding to velocities $V_{i}$ = 100 m/s, 200 m/s and 300 m/s. Since the differences in $\sigma_{a}$ among the same densities under 100 m/s, 200 m/s and 300 m/s are not significant (as shown in Figure 6), only the curve of Equation (9) corresponding to 200 m/s is presented.

From Figure 17a, it can be observed that both Equations (8) and (9) can characterize the uniaxial constitutive law of the PVC foam under shock. It has a better agreement at higher and lower velocities when the strain rate of the base material is and is not considered, respectively. In the four LCs, the entrapped gas reduces the stress, whereas the strain rate effect of the base material increases the stress. The stress–strain curve under shock is different from that under quasi-static loading and the former is generally below the latter. But, when the strain effect of the base material and the entrapped gas effect are both considered, the stress–strain curve under shock is fully above the quasi-static curve when $\rho^{*}/\rho_{s}$ = 0.07, but only at lower impact velocities when $\rho^{*}/\rho_{s}$ = 0.14 or 0.21. It can also be found that the influence of the strain rate effect of the base material and the entrapped gas effect is more significant for lower density foam.

## 6. Conclusions

This paper presents FEA of the compressive responses of closed-cell PVC foam under shock based on a cell-based model. With the CEL method and a strain rate dependence material model for the PVC being adopted, the influences of the strain rate effect of the base material and the entrapped gas effect on the mechanical properties, such as stress, strain, velocity and shock front position, were investigated. The main conclusions are as follows:

(1) The entrapped gas mainly affects the shock strain *ε_b_*, whereas the strain rate effect of the base material mainly affects the shock stress *σ_b_*. The strain rate effect of the base material is more significant overall.
(2) The impact speed has a significant effect on the velocity field, but the relative density, which is changed by the average wall thickness, has a negligible effect.
(3) When the strain rate of the base material is considered, neither the quasi-static yield strength nor the nominal strain rate cannot be used as the respective representative values ahead of the shock front. Therefore, this study proposes a new definition of the nominal strain rate, with which the calculated stress agrees well with the FEA predicted value at the supporting end.
(4) The higher the impact speed, the greater the stress fluctuation is at the impact end. But, the degree of stress fluctuation at the supporting end is not significantly affected by the impact speed.
(5) A constitutive law includes the strain rate of the base material, and the entrapped gas effect was proposed for the PVC material.

In summary, the current research clarifies the mechanisms of the dynamic responses of the PVC foams under shock and thus is helpful for the design and optimization of the PVC foams subjected to uniaxial impact. For example, the threshold impact speed to promote the shock can be predicted by the constitutive law. However, in a sandwich member comprised of fiber-reinforced plastic sheets and foam cores, where the PVC foam is most widely used, uniaxial load is seldom, if any. So, more work should be carried out to study the tri-axial dynamic behavior of the PVC foams, which is the next step objective.

## Figures and Tables

**Table 1:** Basic parameters of the entrapped gas [36].

Density (kg/m^3^)	Ambient Pressure (MPa)	Gas Constants (KJ/kg·K)	Specific Heat (KJ/kg·K)
1.293	0.1	0.287	0.717

**Table 2:** $V_{r}$ and *S* for the foams of different relative densities in the four LCs.

$\rho^{*}/\rho_{s}$ = 0.07	$V_{r}$ (m/s)	S	$\rho^{*}/\rho_{s}$ = 0.14	$V_{r}$ (m/s)	S
LC1	10.69	1.004	LC1	10.39	1.008
LC2	15.58	1.001	LC2	22.92	0.9731
LC3	40.46	0.9510	LC3	36.96	0.946
LC4	45.62	0.9701	LC4	40.93	0.9605
$\rho^{*}/\rho_{s}$ = 0.21	$V_{r}$ (m/s)	S			
LC1	13.90	0.994			
LC2	25.54	0.9639			
LC3	35.19	0.941			
LC4	35.39	0.9684			

## Data Availability

The data presented in this study are available on request from the corresponding author.
